# A White Spot Around the Fissula Ante Fenestrum: A New Diagnostic Indicator for Otosclerosis

**DOI:** 10.3390/jcm14030913

**Published:** 2025-01-30

**Authors:** Tatsuya Yamasoba, Tsukasa Uranaka, Hajime Koyama, Akinori Kashio

**Affiliations:** 1 Department of Otolaryngology and Head and Neck Surgery, Faculty of Medicine, University of Tokyo, Tokyo 113-8655, Japan; uranaka-tky@umin.ac.jp (T.U.); hakoyama-tky@umin.ac.jp (H.K.); kashioa-oto@h.u-tokyo.ac.jp (A.K.); 2 Department of Otolaryngology, Tokyo Teishin Hospital, Tokyo 102-8798, Japan; 3 Department of Otolaryngology, Toranomon Hospital, Tokyo 105-8470, Japan

**Keywords:** middle ear, stapes, oval window, endoscope

## Abstract

**Background/Objectives**: Since we started endoscopic stapes surgery, we have frequently noticed a white spot (WS) with a clear boundary on the bone surface around the fissula ante fenestrum (FAF) in otosclerosis cases. We investigated the significance of this surgical finding. **Methods**: We enrolled 27 cases of otosclerosis and 28 control cases of conductive hearing loss due to pathologies other than otosclerosis, both operated on endoscopically at the University of Tokyo Hospital. We retrospectively reviewed surgical videos to determine whether WS was present or absent. We examined the incidence of WS in otosclerosis cases and the controls and also in cases of otosclerosis, the presence or absence of WS was compared with the preoperative hearing level, preoperative air–bone gap, vasodilatation on the promontory, and a low-density area on high-resolution computed tomography (HRCT). **Results**: WS was present in 11 (41%) of 27 cases of otosclerosis but none in 28 control cases. There were no significant differences in patients’ age and gender, the incidence of vasodilatation on the promontory, preoperative hearing level, or preoperative air–bone gap between otosclerosis cases with and without WS. Although a hypodense focus anterior to the oval window was more frequently present on HRCT in otosclerosis cases with WS (82%) than those without WS (56%), the difference in the incidence failed to reach significance (*p* = 0.10). **Conclusions**: We observed WS around the FAF only in cases of otosclerosis, indicating that WS is unique in otosclerosis. WS did not correlate with vasodilatation on the promontory, preoperative hearing level, or air–bone gap. A hypodense focus anterior to the oval window on HRCT tends to be more common in otosclerosis cases with WS.

## 1. Introduction

Otosclerosis is a localized bone disease that only affects the endochondral bone of the otic capsule in humans. The foci of disordered bone resorption, new bone deposition, vascular proliferation, and/or connective tissue stroma are present in histology. Four stages are generally recognized as occurring in the development and progression of otosclerotic lesions: (1) Destruction of enchondral bone with the formation of resorption spaces that contain highly cellular fibrous tissue. (2) Formation of mucopolysaccharide and osteoid deposits within the fibrous collagen of the resorption spaces, leading to the production of immature basophilic bone. (3) Repetition of the remodeling process of resorption and new bone formation through several generations with the development of a more mature acidophilic bone with a laminated matrix. (4) Formation of a highly mineralized acidophilic bone with a mosaic-like appearance. The otosclerotic process may become quiescent at any time or become reactivated [1]. Nylen [2] examined the incidence of the otosclerotic foci in the temporal bones of seventy-four cases with typical cases of otosclerosis and found that the localization of foci was in the oval window region in 90%, which was accompanied by stapes ankylosis in 50%, and in the round window region in 40%. The cochlear capsule was involved in 35%, the internal auditory canal region in 30%, and the semi-circular canal capsule in 15%.

Otosclerosis is categorized into fenestral and retrofenestral types based on the extent of involvement, and into spongiotic (active) or sclerotic (inactive) types based on the phase of the disease [1]. Otosclerosis may cause conductive, mixed, or, rarely, pure sensorineural hearing loss. The otic capsule anterior to the oval window, around the fissula ante fenestrum (FAF), is the most common site of involvement. If the lesion is confined to this area, it is called fenestral otosclerosis. The involved site can be seen as a lucent or hypodense area on high-resolution computed tomography (HRCT) because of the resorption of the enchondral bone during the spongiotic (active) phase [3,4,5]. As the disease progresses to the inactive or sclerotic phase, these lesions are remineralized and may become indistinguishable on HRCT from the normal otic capsule [3,4,5]. Otosclerotic foci are usually larger in volume than the bone they replace [1].

Diagnosis of otosclerosis is made based on history, physical examination, and audiometric testing and is supported by imaging studies. Acquired conductive hearing loss accompanied by the normal tympanic membrane is one of the indicators of otosclerosis. Carhart’s notch, defined as a bone conduction threshold elevation of 2 kHz, has been a well-known indicator of stapes fixation. However, the elevation in bone conduction thresholds between 1 and 4 kHz can be caused by various factors that affect the conductive mechanism of the middle ear, including a detachment of the incudostapedial joint and malleus or incus fixation [6]. The Schwartze sign, which is named after the German otologist Hermann Schwartze, is a characteristically reddish discoloration of the promontory seen during an otoscopic examination. This discoloration is the result of the increased vascularization on the promontory and occurs in fewer than 10% of patients with otosclerosis [7].

HRCT is the technique of choice to diagnose otosclerosis or other pathologies and to evaluate coexisting diseases and surgical anatomy [3]. On HRCT, otosclerosis appears as a lucent or hypodense focus within the otic capsule, most commonly anterior to the oval window. Other radiological findings in otosclerosis include a thickened footplate, narrowed oval window or round window niche, and the double ring sign (a hypodense lesion surrounding the cochlea) [5,8,9]. Wegner et al. [10] performed a systemic review of the diagnostic value of HRCT imaging in diagnosing otosclerosis, including seven studies characterized by a moderate-to-high relevance and moderate-to-low risk of bias for data extraction, and found that in studies with high disease prevalence, positive and negative post-test probabilities were 99% and between 51% and 67%, respectively, and the overall reported sensitivities ranged from 60% to 95% when including one study with a low prevalence of disease (9%) and positive and negative post-test probabilities of 23% and 3%, respectively.

Although there is no widely accepted radiological classification of otosclerosis, several different staging systems have been proposed. Valvassori initially proposed a grading system for cochlear otosclerosis based on disease location and progression [11]. Shin et al. [12] divided the subjects into fenestral and pericochlear groups and subdivided the pericochlear group into one with an extension to the cochlear endosteum and the other without it. Kiyomizu et al. [13] classified fenestral disease into group A with no pathologic CT findings, group B1 with demineralization localized to the FAF, group B2 with demineralization extending from the anterior region of the oval window toward the cochleariform process, group B3 with extensive demineralization surrounding the cochlea, and group C with thick anterior and posterior calcified plaques. Rotteveel et al. [14] described a classification system based on the appearance of the involvement of the otic capsule: type 1 is only a fenestral involvement; type 2 is a cochlear involvement (with or without a fenestral lesion), divided into types 2a (“double ring effect”), type 2b (narrowed basal turn), and type 2c (“double ring effect” and narrowed basal turn). Type 3 is a severe cochlear involvement (unrecognizable otic capsule).

Symons and Fanning [15] proposed a CT grading system for otosclerosis: grade 1: fenestral lesions, either spongiotic or sclerotic lesions, evident as a thickened stapes footplate, and/or decalcified, narrowed, or enlarged round or oval windows; grade 2: patchy localized cochlear lesions (with or without fenestral involvement) localized around the basal cochlear turn (grade 2A), the middle/apical turns (grade 2B), or both turns (grade 2C); and grade 3: diffuse confluent cochlear lesions of the otic capsule (with or without fenestral involvement). Grade 3 is distinguished from grade 2C by the diffuse confluent involvement of the entire cochlea, whereas grade 2C has patchy focal involvement of the whole cochlea. Veillon [16] classified axial CT images of the petrous bone in patients with otosclerosis: type 0, normal; type 1a, isolated involvement of the footplate, which is thickened (>0.6 mm) and hypodense; type 1b, anterior fenestral hypodensity ≤ 1 mm; type 2, anterior fenestral hypodensity > 1 mm, without contact of the focus with the cochlear endosteum; type 3, anterior fenestral hypodensity > 1 mm and with the contact of the focus with the cochlear endosteum; type 4A, anterior labyrinthine hypodensities around the cochlea; and type 4B, posterior labyrinthine hypodensities around the semicircular canals or the vestibule. A recent study [17] involving a total of 87 patients and 97 operated ears showed that the Symons–Fanning classification was associated with preoperative bone conduction and air conduction thresholds and postoperative bone conduction thresholds with an increase in thresholds with higher radiological stages, the Veillon classification was related to postoperative air conduction thresholds, with an increase in the thresholds with increasing radiological stages, and both classifications were associated with a decrease in the rates of postoperative air conduction thresholds ≤30 dB with higher radiological stages.

Although conductive or mixed hearing loss accompanied by the normal eardrum, Carhart’s notch, Schwartze sign, and abnormal HRCT findings described above all suggest the presence of otosclerosis, the diagnosis is finally made during surgical exploration by confirming the fixation or marked loss of mobility of the stapes footplate. This diagnosis process during surgery is not difficult in most cases of otosclerosis; however, it may not be so easy in some cases, especially when the facial nerve overhangs downward toward the stapes, limiting the view of the stapes footplate. In such a case, additional surgical findings would be helpful in diagnosing otosclerosis.

A recent development of endoscopic technology makes endoscopic ear surgery easy to perform because an endoscope provides a wider and clearer field of view at optimal magnification. Endoscopic stapes surgery has become a preferable alternative to conventional microscopic stapedotomy as it yields similar hearing outcomes and lower pain scores [18]. We recently started endoscopic stapes surgery and have frequently noticed a white spot (WS) with a clear boundary on the bone surface around the FAF in cases of otosclerosis. In the current study, we examined the incidence of WS in cases of otosclerosis and those of conductive hearing loss due to other pathologies. We also compared patients’ age and gender, the presence of vasodilatation on the promontory, preoperative hearing level, preoperative air–bone gap, and the incidence of low-density area anterior to the oval window on HRCT in otosclerosis cases with and without WS.

## 2. Patients and Methods

### 2.1. Patients

We enrolled 27 cases of 24 patients with otosclerosis and 28 cases of 28 patients with conductive hearing loss due to pathologies other than otosclerosis, such as a congenital ossicular anomaly, traumatic ossicular dislocation, and congenital cholesteatoma. All patients underwent pure-tone audiometry, tympanogram and acoustic reflex audiometry, a speech perception test, and a tuning fork test (Rinne’s and Weber’s test). HRCT was also performed to determine the differential diagnosis and the eligibility for stapes surgery or tympanoplasty. We used the guidelines of the American Academy of Otolaryngology-Head and Neck Surgery (https://www.entnet.org/wp-content/uploads/files/Stapedectomy-CI%20Updated%208-7-14.pdf (accessed on 28 January 2025)) as the criteria for the indication of stapes surgery.

The diagnosis of otosclerosis and other pathologies was confirmed during surgery by examining middle ear spaces and the continuity and mobility of the ossicles. Three patients with otosclerosis underwent stapes surgery bilaterally. Both groups’ patients showed good middle ear aeration and were operated on endoscopically between September 2019 and March 2023 at the University of Tokyo Hospital. All patients had no intraoperative or postoperative complications, and according to the guideline for reporting hearing results in the middle ear and mastoid surgery (2010) by the Japan Otological Society (https://www.otology.gr.jp/common/pdf/mastoid2010.pdf, in Japanese (accessed on 28 January 2025)), all cases were judged to achieve successful hearing improvement.

This study was approved by the Regional Ethical Standards Committee of the Faculty of Medicine at the University of Tokyo (application number 2487) and was conducted by the tenets of the Declaration of Helsinki. Informed consent was obtained through an opt-out procedure.

### 2.2. Evaluation of Surgical Video and Patients’ Hearing

We retrospectively reviewed the surgical video and medical records of these patients. The presence or absence of WS around the oval window was evaluated independently by three to four experienced otology surgeons. The final decision on the presence of WS was made when the results of all the evaluations were agreed upon. Before stapes surgery, we measured air conduction thresholds from 0.125 to 8 kHz and bone conduction thresholds at 0.5, 1, 2, and 4 kHz. Audiometric pure-tone air and bone conduction thresholds averaged over four frequencies (0.5, 1, 2, and 4 kHz) were calculated.

### 2.3. HRCT Evaluation

HRCT images were obtained with either a 4-detector row CT scanner (0.5 mm width, 120 kV, 200 mA; Toshiba Aquilon 16, Ohtawara, Japan) or a 320-detector row CT scanner (0.5 mm width, 120 kV, 100 mA; Toshiba Aquilon ONE6, Ohtawara, Japan), with a reconstruction spacing of 0.1 mm. Images were evaluated by 2 radiologists and 3 otologists at a GE Medical Systems Advantage Workstation.

### 2.4. Statistical Analysis

Statistical analysis was performed using Fisher’s exact test to compare the incidence of WS and gender differences between the otosclerosis and control cases and to compare the gender differences, incidence of vasodilatation, and CT abnormalities between otosclerosis cases with and without WS. Welch’s *t*-test was used to compare age between the otosclerosis and control cases and postoperative hearing thresholds and air–bone gaps between otosclerosis cases with and without WS. The Mann–Whitney U test was used to compare the age of otosclerosis cases with and without WS, as the datasets were not normally distributed using the Shapiro–Wilk test.

## 3. Results

The patients ranged in age from 16 to 73 years (54 ± 17 years) in the otosclerosis group and from 3 to 81 years (50 ± 28 years) in the control group. There were 11 males and 13 females in the otosclerosis group and 12 males and 16 females in the control group. There were no significant differences in age and gender between the groups.

**Figure 1:**
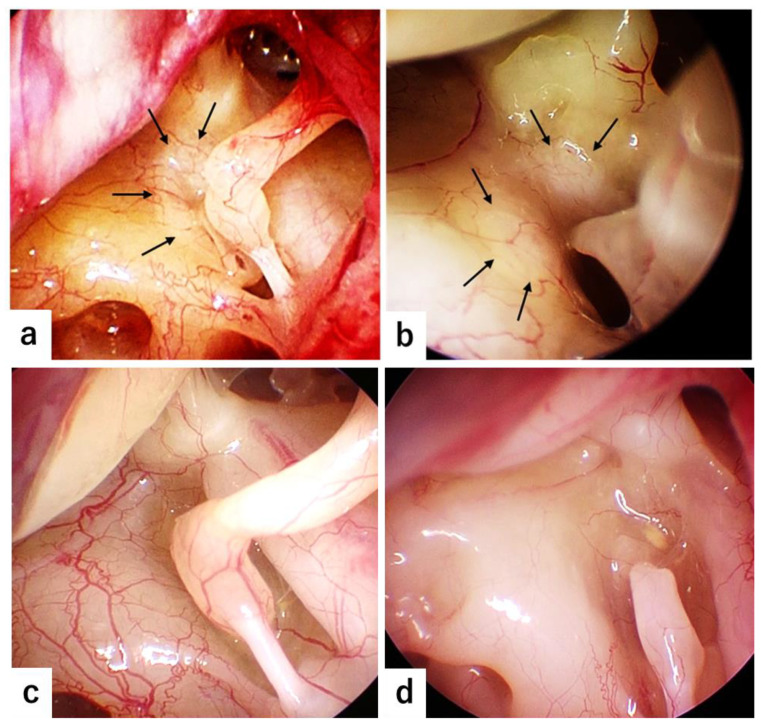
Typical video-captured images in two otosclerosis cases with a white spot (WS) with a clear boundary on the bone surface anterior to the oval window (**a**,**b**), in an otosclerosis case without WS (**c**), and in a case with congenital ossicular anomaly (**d**). Vasodilatation on the promontory is minimal in three otosclerosis cases and absent in a congenital ossicular anomaly case. (**a**) WS is widely present anterior to the oval window, surrounding the anterior half of the stapes footplate. (**b**) Two WSs are observed anteroinferior and anterosuperior to the oval window. Arrows indicate WS.

The evaluation results regarding the presence or absence of WS were consistent in all cases, as WS was clearly observed in the endoscopic evaluation when present. There were no equivocal cases that made split judgments. WS was observed in 11 (41%) of 27 cases of otosclerosis but not in 28 control cases, with the difference in the incident being statistically significant (*p* = 0.0001). In three patients who underwent stapes surgery bilaterally, WS was present in both ears in two patients and only in one ear in one patient. Typical video-captured images of WS are shown in Figure 1.

Table 1 shows the presence or absence of WS, patient age and gender, presence or absence of vasodilatation on the promontory, preoperative hearing level, preoperative air–bone gap, and CT findings in patients with otosclerosis. The age of 11 otosclerosis cases of 9 patients with WS was 52 ± 11 years and that of 16 cases of 16 patients without WS was 56 ± 17 years. There were five males and four females in patients with WS and seven males and nine females in those without WS. Marked hypervascularity or vasodilatation was observed on the promontory in one case with WS and three cases without WS. In the remaining cases, the vasodilatation was not evident or minimal if present. The preoperative hearing thresholds and air–bone gaps were 54.5 ± 19.5 dB and 33.5 ± 9.5 dB, respectively, in 11 otosclerosis cases with WS and 66.6 ± 14.2 dB and 3.1 ± 9.9 dB in 16 cases without WS. Patient age, gender, the incidence of vasodilatation on the promontory, the preoperative air conduction threshold, and preoperative air–bone gap were not significantly different between otosclerosis cases with and without WS (*p* = 0.20, *p* = 0.70, *p* = 0.62, *p* = 0.09, and *p* = 0.81, respectively).

On HRCT, a hypodense focus anterior to the oval window was observed in 16 of 27 (59%) otosclerosis cases: 9 of 11 (82%) cases with WS and 7 of 16 (44%) cases without WS. Although the incidence of the hypodense focus identified was greater in cases with WS than those without WS, the difference failed to reach statistical significance (*p* = 0.10). A hypodense area surrounding the otic capsule (double ring sign) was present only in two cases without WS.

## 4. Discussion

We detected WS in 11 (41%) of 27 cases of otosclerosis but not in 28 control cases of conductive hearing loss due to other pathologies. This finding is considered disease-specific and therefore a distinct indicator of diagnosing otosclerosis. The presence of WS was not associated with patients’ age and gender, hypervascularity on the promontory, preoperative hearing level, or preoperative air–bone gap. A hypodense area anterior to the oval window appeared to be more frequently observed on HRCT in otosclerosis cases with WS (82%) than those without WS (44%), although the difference in the incidence of this CT abnormality did not reach statistical significance.

The small number of cases reviewed in our study does not allow us to draw any conclusions regarding the incidence of WS in clinically diagnosed cases of otosclerosis. Asian people are known to have a lower incidence of otosclerosis than Caucasian people. Gordon reported that the incidence of otosclerosis in Japanese people was approximately 1/50 of that in Caucasian people [19]. Further, an aggressive form of otosclerosis, named obliterative otosclerosis, has been reported to occur in 4.1~11.4% of Caucasian people [20,21,22], whereas it is seldomly observed in Japanese people [23], suggesting that cases of large otosclerosis foci are more common in Caucasian people than in Japanese people. Therefore, WS may be recognized more frequently in Caucasian people. Further studies are needed to determine the incidence of WS in different racial groups.

It is widely acknowledged that HRCT exhibits high specificity and positive predictive value when diagnosing otosclerosis [10,24,25]. The prevalence of pathological findings on CT, including demineralized and sclerotic lesions, may vary among reports, but the most common site was always located anterior to the oval window, including the FAF [26,27,28,29]. In the current study, a hypodense focus anterior to the oval window was observed in 16 of 29 (55%) otosclerosis cases. This incidence of abnormal CT findings was similar to that in previous reports from Japan. Kinomizu et al. reported that there was demineralization localized in the region of the FAF in 21 ears (25.6%), demineralization extending towards the cochleariform process from the anterior region of the oval window in 16 ears (19.5%), and extensive demineralization surrounding the cochlea in 7 ears (8.5%) in 82 ears of 44 patients with surgically confirmed otosclerosis [13]. We previously evaluated CT findings in 42 ears with otosclerosis and observed otospongiotic lesions in 21 ears (50%), of which the focus was localized in the region of the FAF in 14 ears and not only in the region of the FAF but around the cochlea or along the internal auditory meatus in 6 ears. It should be noted that these reported incidences of CT abnormality in Japanese cases of otosclerosis are much lower compared to those reported in recent studies from Western countries. For example, Lagleyre et al. [26] evaluated HRCT findings in 209 ears of 200 patients with otosclerosis before surgery and reported that 84.2% of cases were classified positive, 8.6% doubtful, and 7.2% negative. Virk et al. [27] reviewed the contemporary English medical literature from 1990 to 2013 via MedLine using terms such as imaging, otosclerosis, stapes surgery, and computed tomography and reported that multidetector scanners demonstrated a sensitivity and specificity of over 90%. We mentioned above that the incidence of otosclerosis and aggressive lesions such as obliterative otosclerosis is much less in Japanese people than in Caucasian people. Similarly, otosclerotic foci may be smaller in Japanese people than in Caucasian people, resulting in the lower detectability of CT abnormalities.

In the current study, a hypodense focus anterior to the oval window was observed in 82% of otosclerosis cases with WS and 44% of those without WS, suggesting that a hypodense focus anterior to the oval window on HRCT may be more common in cases with WS, although the difference failed to reach statistical significance. The extension and location of otosclerotic bones can vary among cases of otosclerosis, and otosclerotic foci may be exposed widely to the surface of the middle ear spaces in some cases but not in others [1]. It seems that an otosclerotic focus is larger when it is exposed to the middle ear spaces and appears as WS; a hypodense focus anterior to the oval window may be more likely to be detected in such cases.

The potential mechanisms underlying the observed white spots are unknown. Histologically, active otosclerosis is characterized by large pseudovascular spaces that result from the resorption of the enchondral bone around blood vessels, as well as high cellularity, osteoclasts, and deposition of the woven bone. In contrast, inactive otosclerosis has few pseudovascular spaces, low cellularity, no osteoclasts, and predominantly lamellar bone [1,4,30]. Quesnel et al. [31] measured CT density on 78 human temporal bone specimens (31 with otosclerosis and 47 controls) that had undergone high-resolution multidetector CT before histologic processing. They applied a scale for grading otosclerosis histologically based on the percentage of pseudovascular spaces to capture the essence of active versus inactive otosclerosis: grade 0, no otosclerosis; grade 1, pseudovascular space is <25% (inactive otosclerosis); grade 2, 25–50% (mixed area of active and inactive otosclerosis); and grade 3, >50% (active otosclerosis). As a result, they found that the histologic grade of otosclerosis was inversely related to CT density at the most common site of otosclerosis predilection, anterior to the oval window and also adjacent to the anterior portion of the basal turn of the cochlea. Although not statistically significant, we found a trend that WS was prevalent in otosclerosis cases showing a hypodense focus on HRCT examination. Therefore, this color change in the bone surface is likely to reflect histologically active otosclerosis characterized by large pseudovascular spaces and the deposition of the woven bone.

The diagnosis of otosclerosis is finally made during surgery when stapes fixation or a marked reduction in stapes mobility is confirmed. This diagnosing process is not difficult in most cases of otosclerosis but may not be easy when the facial nerve overhangs downward toward the stapes, thereby limiting the view of the footplate. If the stapes crura are loosely connected to the footplate and easily mobile, it may also make it difficult to confirm the fixation or the reduced mobility of the footplate. Epitympanic ossicular fixation of the incus, the malleus, or both can occur without prior chronic otitis media and is more frequent in ears with otosclerosis than in the general population [32]. If a surgeon diagnoses epitympanic fixation unaccompanied by otosclerosis by mistake because of the relatively mobile stapes superstructure that is loosely connected to the fixed footplate, the incus or incus long process may be removed to place a columella on the stapes head, which makes it impossible to hook a piston onto the incus long process. In such a case, the presence of WS is quite helpful in confirming the diagnosis of co-existing otosclerosis.

This study has some limitations. First, the sample size was small and insufficient to analyze the correlations between WS and other factors such as CT findings. A larger cohort is needed to draw meaningful conclusions regarding the incidence and significance of WS in otosclerosis. Second, because this was a retrospective study, it remains possible that very small WSs were missed that could have been detected by more rigorous observation.

## 5. Conclusions

We observed WS around the FAF in 11 (41%) of 27 cases of otosclerosis but none in 28 control cases, indicating that WS is unique for otosclerosis and helpful in diagnosing it during surgery. WS did not correlate with patient age or gender, vasodilatation on the promontory, preoperative hearing level, or air–bone gap. A hypodense focus on HRCT tends to be more common in otosclerosis cases with WS. Considering that otosclerotic foci are likely to be smaller and less aggressive in Japanese people than in Caucasian people, further studies are needed to determine the incidence of WS and its relationship with CT abnormalities in other racial groups.

## Figures and Tables

**Table 1:** Patients’ age and gender, the presence of vasodilatation, hearing level, air–bone gap (ABG), and CT findings in cases of otosclerosis with and without a white spot (WS) on the bony surface around the fissula ante fenestrum (FAF). PTA: pure-tone average; RWN: round window niche. ^#^, ^+^, and * indicate the same patients.

WS	Age (yr)	Gender	Vasodilatation	Preoperative PTA	Preoperative ABG	Hypodense Focus at FAF	Double Ring Sign
Yes	73	M		98.75	43.75		
Yes	44 ^#^	M		55	41.25	Yes	
Yes	22	M	Promontory	56.25	40	Yes	
Yes	51	F		20	11.25		
Yes	44 ^#^	M		51.25	36.25	Yes	
Yes	56 ^+^	M		52.5	37.5	Yes	
Yes	53	M		45	30	Yes	
Yes	72	F		75	41.25	Yes	
Yes	44 *	F		53.75	26.25	Yes	
Yes	55	F		45	26.25	Yes	
Yes	45 *	F		47.5	35	Yes	
	72	M	RWN	80	32.5	Yes	Yes
	70	M		96.25	33.75		
	64	F		78.75	36.25		
	16	F		93.75	57.5		
	21	F		56.25	36.25	Yes	
	62	M	Promontory	68.75	40		
	68	M		62.5	28.75	Yes	
	65	M		67.5	31.25		
	56 ^+^	M		65	45		
	38	M		52.5	37.5	Yes	Yes
	72	F		53.75	21.25	Yes	
	45	F	Promontory	65	16.25	Yes	
	60	F		62.5	23.75		
	62	F		46.25	41.25		
	64	F		61.25	27.5		
	58	F	Promontory	55	37.5	Yes	

## Data Availability

The data supporting this study’s findings are available from the corresponding author upon reasonable request.
